# *Rheb1* deletion in myeloid cells aggravates OVA-induced allergic inflammation in mice

**DOI:** 10.1038/srep42655

**Published:** 2017-02-22

**Authors:** Kai Li, Yue Zhang, Kang Yan Liang, Song Xu, Xue Juan Zhou, Kang Tan, Jun Lin, Xiao Chun Bai, Cui Lan Yang

**Affiliations:** 1 Department of Cell Biology, School of Basic Medical Science, Southern Medical University, Guangzhou, Guangdong, China

## Abstract

The small GTPase ras homolog enriched in brain (*Rheb*) is a downstream target of tuberous sclerosis complex 1/2 (*TSC1/2*) and an upstream activator of the mechanistic target of rapamycin complex 1 (mTORC1), the emerging essential modulator of M1/M2 balance in macrophages. However, the role and regulatory mechanisms of *Rheb* in macrophage polarization and allergic asthma are not known. In the present study, we utilized a mouse model with myeloid cell-specific deletion of the *Rheb1* gene and an ovalbumin (OVA)-induced allergic asthma model to investigate the role of *Rheb1* in allergic asthma and macrophage polarization. Increased activity of *Rheb1* and mTORC1 was observed in myeloid cells of C57BL/6 mice with OVA-induced asthma. In an OVA-induced asthma model, *Rheb1*-KO mice demonstrated a more serious inflammatory response, more mucus production, enhanced airway hyper-responsiveness, and greater eosinophil numbers in bronchoalveolar lavage fluid (BALF). They also showed increased numbers of bone marrow macrophages and BALF myeloid cells, elevated M2 polarization and reduced M1 polarization of macrophages. Thus, we have established that *Rheb1* is critical for the polarization of macrophages and inhibition of allergic asthma. Deletion of *Rheb1* enhances M2 polarization but decreases M1 polarization in alveolar macrophages, leading to the aggravation of OVA-induced allergic asthma.

Asthma is a chronic inflammatory lung disease that is characterized by airway hyper-responsiveness to allergens, airway remodeling, and increased mucus secretion^1^. T-helper type 2 (Th2) cells are dominant in the airway reactions and Th2 cytokines such as IL-4, IL-5 and IL-13 play a pivotal role in the pathophysiology of asthma^2^, and are involved in the differentiation of alternatively-activated (M2) macrophages^3^. These macrophages can generate several proinflammatory factors, such as chemokines, chitinase-like molecules and ‘found in inflammatory zone 1′ (FIZZ1, also known as Relm-α), which all contribute to the inflammation and remodeling of airways in asthma^4^,^5^. Markers of M2 macrophages correlate with the severity of allergic airway disease in humans and mice, suggesting that M2 macrophages contribute to the disease^6^. M1 macrophages are differentiated by interferon (IFN)-γ and lipopolysaccharide (LPS) derived from γ-negative bacteria in both mice and humans^7^. These macrophages release inflammatory cytokines and chemokines (IL-12, IL-6, TNF-α, and CXCL10, CCL3), produce high levels of nitric oxide, and play an important protective role against intracellular pathogens. In short, the polarization status of macrophages plays a vital role in asthma^8^,^9^,^10^, but the relevant mechanism by which M2 macrophages reduce the Th2 cell response has not been fully investigated.

It is widely known that mechanistic target of rapamycin (mTOR) is a conserved Ser/Thr kinase consisting of at least two distinct multi-protein complexes, mTOR complex 1 (mTORC1) and mTOR complex 2 (mTORC2)^11^. Previous studies have shown that mTORC1 plays a critical role in macrophage polarization^12^,^13^. Tuberous sclerosis complex 1 (*TSC1*) and tuberous sclerosis complex 2 (*TSC2*) form a complex which regulates metabolism and energy homeostasis via inhibition of mammalian target of rapamycin complex 1 (mTORC1)^14^. Loss-of-function mutations of *TSC1* or *TSC2* result in elevated activity of mTORC1, which leads to increased cell growth and proliferation^15^. A recent study using mice with myeloid-specific deletion of *TSC1* found that *TSC1*-deficient mice are highly resistant to M2-polarized allergic asthma, and identified a key role for *TSC1* in orchestrating macrophage polarization via mTOR-dependent and independent pathways^16^. However, the alteration of mTOR activity and the function of endogenous inhibition of mTORC1 in asthma and macrophage polarization have not been elucidated.

*Rheb* (ras homolog enriched in brain) belongs to the ras superfamily of GTPases, which is essential for development of both flies and mice, as well as being a potent activator of mTORC1^17^,^18^. Two *Rheb* family members, *Rheb1* and *Rheb2*, are found in mammals, among which *Rheb1* is found to be the essential isoform in mice and appears to be the dominant regulator of mTORC1^19^,^20^. *Rheb1* can bind directly to the active kinase domain of mTOR, but *Rheb1* mutants with nucleotide-deletion trap mTOR in a catalytically-inactive state^21^. Nevertheless, *Rheb*-GTP targets other than mTOR, such as FKBP38 (FK506-binding protein 38) and/or PLD1 (phospholipase D1), may also contribute to mTOR activation^22^,^23^. *Rheb1* acts epistatically to exert an inhibitory effect of the *TSC1-TSC2* heterodimer on mTORC1 signaling, and the relationship is explained by the finding that *TSC* is an activator of *Rheb1* GTPase activity^24^. However, the role of *Rheb1* in regulation of allergic asthma and macrophage polarization is still not fully understood.

In this study, we found increased activity of *Rheb1* and mTORC1 in myeloid cells of C57BL/6 mice with ovalbumin (OVA)-induced allergic inflammation. We utilized a mouse model with myeloid-specific deletion of *Rheb1* to study the role of *Rheb1* in OVA-induced allergic asthma. We found that *Rheb1*-knock out (KO) mice are more susceptible to OVA-induced allergic asthma than wild-type (WT) mice. Further, we noticed that *Rheb1*-KO macrophages show a marked enhancement of M2 polarization and an obvious deficiency of M1 polarization. Accordingly, we speculate that *Rheb1* may influence the extent of inflammatory reaction in a mouse model of OVA-induced allergic asthma by participating in the regulation of macrophage polarization. Thus, we propose that *Rheb1* may be a new target for treatment of allergic asthma.

## Results

### Increased activity of *Rheb1* and mTORC1 is found in BALF cells of C57BL/6 mice with OVA-induced allergic inflammation

**Figure 1:**
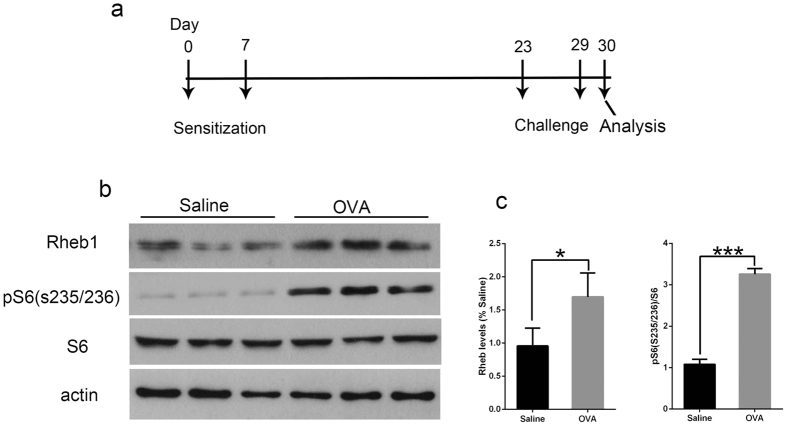
mTORC1 activity was elevated in OVA-induced allergic asthma mice compared to saline-treated mice. (**a**) Experimental protocol of the study, n ≥ 5 per group. (**b**) Western blot analysis of cells from BALF of each group, n = 3 per group. (**c**) Quantitative analysis of protein levels of *Rheb1* and pS6(s235/236) in BALF of each group. *P < 0.05; ***P < 0.001.

To observe the activity of *Rheb1* and mTORC1 in allergic asthma, C57BL/6 mice were separated into two groups: in the asthma group mice were treated by intraperitoneal injection (i.p.) of OVA emulsified in aluminum hydroxide gel at day 0 and day 7, then they were challenged with OVA inhalation for 7 days from day 23 to day 29 (Fig. 1a), while mice in the control group were sensitized and challenged with saline. On day 30, all of the mice were sacrificed, and BALF from the mice in both groups was collected and centrifuged to obtain cells which were lysed in lysis buffer. Western blot analysis showed that *Rheb1* and mTORC1 downstream protein pS6 (s235/236) were both much more highly expressed in the asthma group than in the control group (Fig. 1b,c). Thus, we can preliminarily conclude that *Rheb1* expression and mTORC1 activity are both markedly increased in the OVA-induced allergic asthma model group compared with the control group, suggesting that mTORC1 and *Rheb1* may play a vital role in regulating allergic asthma.

### Absence of *Rheb1* inhibits mTORC1 signaling in myeloid cells

**Figure 2:**
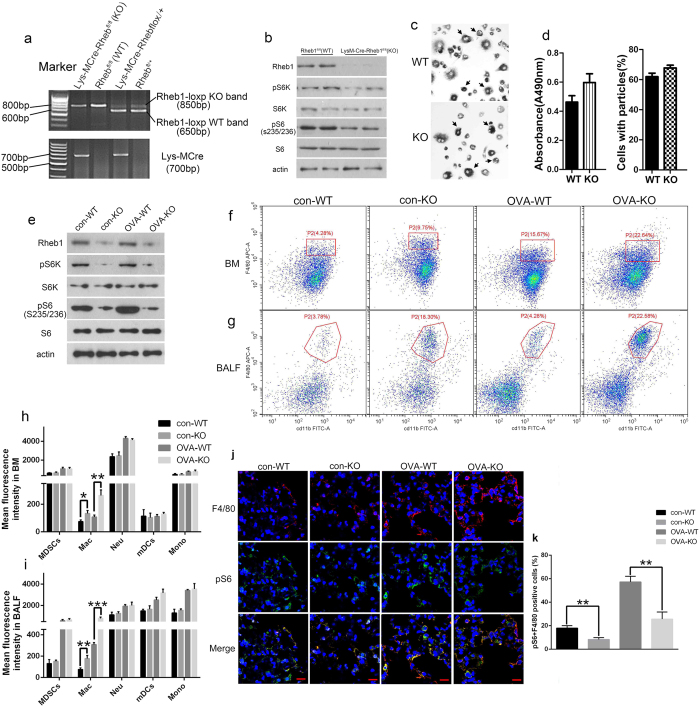
*Rheb1* deletion in myeloid cells inhibited mTORC1 activity in mice. (**a**) Representative results of PCR genotyping using DNA from cells of mouse tails. Mice with the genotype Lys-MCre-*Rheb1*flox/flox were defined as KO and those with *Rheb1*flox/flox as WT. (**b**) Western blot analysis of BMDMs from *Rheb1*^flox/flox^ and LysM-Cre-*Rheb1*^flox/flox^ mice (n = 3). β-actin level was used as the internal reference. (**c**) Macrophage phagocytosis detection, arrows showed the uptake of Indian ink (400X). (**d**) The optical density of neutral red (490 nm) (left) and the percentage of cells with ink particles (%) (right). (**e**) Western blot analysis of sorted alveolar macrophages in BALF from each group (n ≥ 5). β-actin level was used as the internal reference. (**f**) Flow cytometric analysis of macrophage percentages in bone marrow. (**g**) Flow cytometric analysis of macrophage percentages in BALF. (**h**) Quantification of flow cytometric analysis of different cell types in myeloid cells from BM and BALF (**i**). n = 5, *P < 0.05; **P < 0.01. (**j**) IF analysis of lung tissue from WT and KO mice in both the control and asthma groups (Scale bar = 10 μm). (**k**) Quantitative analysis of F4/80 and pS6 positive cells in IF analysis. n ≥ 3, *P < 0.05; **P < 0.01.

To assess the role of *Rheb1* in allergic asthma, we first generated mice with a *Rheb1* specific knockout by mating floxed *Rheb1* mice with Lys-MCre mice (which express a Cre recombinase under the direction of the Lys-M promoter) (as shown in Fig. 2a, Lys-MCre-*Rheb1*flox/flox mice are referred to as homozygote KO mice and *Rheb1*flox/flox as WT mice). Then *Rheb1-*KO mice at 6 weeks of age were divided into two groups; in the asthma group the mice were sensitized and challenged with OVA to induce allergic inflammation, while the corresponding control group was treated with saline (Fig. 1a); each group contained at least six WT and KO mice. Following sensitization, the lung tissue and cells from BALF and bone marrow of each group were analyzed. Immunoblotting confirmed that *Rheb1* is absent from bone marrow cell-derived macrophages (BMDMs) from KO mice, and the expression of the main downstream proteins of mTORC1, p70S6k and pS6, were also down-regulated in KO mice (Fig. 2b). This suggested that *Rheb1* deletion reduced mTORC1 activity in macrophages, and then we detected macrophage phagocytosis from WT and KO mice by neutral red resorption and ink uptake assay. The Results in Fig. 2c,d indicated no significant differences in macrophages function between WT and mutant cells. Moreover, in sorted alveolar macrophages from BALF after OVA treatment, *Rheb1* deletion and mTORC1 reduction were also evident (Fig. 2e).

In conclusion, we successfully generated mice with *Rheb1*-specific knockout in myeloid cells, and *Rheb1*-KO mice displayed inhibition of mTORC1 activity.

### Absence of *Rheb1* increases the percentages of macrophages and inhibits mTORC1 signaling in macrophages

Myeloid cells comprise several different cell types: inflammatory monocytes, macrophages, neutrophils, dendritic cells (mDCs) and a population functionally identified as myeloid-derived suppressor cells (MDSCs). To identify the influences of *Rheb1* deletion on the composition of myeloid cells, we carried out flow cytometry to analyze the different cell types of myeloid cells in *Rheb1*-KO and WT mice. Compared to WT mice, *Rheb1*-KO mice displayed more cells positive for CD11b and F4/80 in bone marrow, and more BALF cells (Fig. 2f,g), indicating a higher percentage of macrophages in KO mice *in vivo*. In the asthma group, the ratio of macrophages in BALF and among bone marrow myeloid cells was higher than the saline group, and *Rheb1-*KO mice had even higher levels of macrophages compared to WT mice (Fig. 2f,g). However other cell types, including monocytes (CD11b+, B220− and CD3−), neutrophils (CD11b+ and ly6G+), dendritic cells (CD11b+, CD11c+ and B220−), and MDSCs (CD11b+ and Gr-1+) exhibited undetectable changes in both groups, whether in blood (data not shown), bone marrow or BALF, in the saline or asthma group (Fig. 2h,i).

Next, we analyzed the mTORC1 activity of alveolar macrophages from *Rheb1*-KO mice in the asthma group and the control group. The expression of F4/80 and pS6 detected by immunofluorescent (IF) staining showed a decrease in KO mice compared with that in WT mice (Fig. 2j,k), which was observed in both the control and asthma groups. In addition, the expression levels of both F4/80 and pS6 were much higher in the asthma group than in the control group (Fig. 2j,k). Thus, deletion of *Rheb1* increases the percentages of macrophages in myeloid cells and inhibits mTORC1 signaling in macrophages.

### *Rheb1* deletion in myeloid cells aggravates OVA-induced allergic asthma in mice

**Figure 3:**
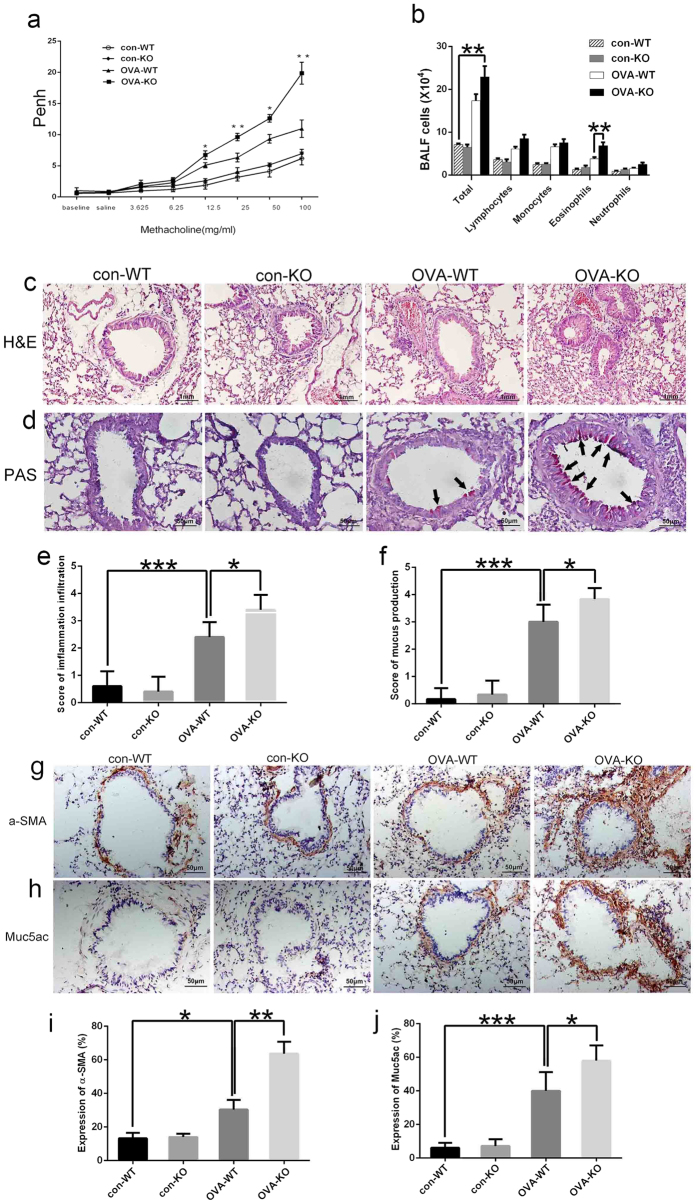
*Rheb1* deletion in myeloid cells aggravated OVA-induced allergic asthma in mice. (**a**) Penh values were collected as described in Materials and Methods. The experiment was repeated three times. (**b**) Differences in the total cell counts in BALF were examined as described in Materials and Methods. (**c**) Lung sections of saline- and OVA-challenged WT mice and *Rheb1*-KO mice were stained with H&E, scale bar is 1 mm. (**d**) PAS staining of lung sections from saline- and OVA-challenged WT mice and *Rheb1*-KO mice (indicated by arrows), scale bar is 50 μm. (**e**). Histopathological changes in lung inflammation and mucus production in an OVA-induced asthma model (**f**) were scored as described in Materials and Methods. (**g**) Immunohistochemical staining of lung sections from each group with α-SMA and Muc5ac (**h**). (**i**) Quantitative analysis of repeated immunohistochemical staining results of α-SMA and Muc5ac (**j**). Scale bar: 50 μm; magnification 400x. Data are shown as mean ± SD; n ≥ 4 per group; *P < 0.05; **P < 0.01; ***P < 0.001.

To investigate the effect of *Rheb1* deletion on OVA-induced asthma, we next examined the role of *Rheb1*-KO in the development of airway hyperactivity (AHR) to methacholine (Mch). No significant difference was found in baseline airway resistance among different groups, but the airway resistance generated by administration of Mch at doses of 6.25 to 100 mg/mL was significantly increased in the OVA-treated groups (Fig. 3a) (P < 0.05). In addition, the OVA-treated *Rheb1*-KO mice showed a significant increase in Penh level at doses from 12.5 to 100 mg/mL compared with WT mice, which was not observed in KO or WT mice with saline treatment (Fig. 3a). In comparison with the saline-treated group, differences in cell number in the BALF from different groups showed that mice treated with OVA displayed an obvious increase in total cell number. In addition, in the OVA-treated group, BALF from *Rheb1*-KO mice contained more eosinophils than that from WT mice (Fig. 3b). As can be seen in Fig. 3c, compared with the saline-challenged controls, the OVA-challenged group displayed typical pathologic features of allergic airway inflammation upon hematoxylin-eosin (H&E) staining, and OVA-challenged mice displayed numerous inflammatory cells infiltrating around the bronchioles, which appeared more numerous in the *Rheb1*-KO mice than in WT mice. As shown in Fig. 3e, OVA-challenged *Rheb1*-KO mice showed a marked increase in inflammation score compared with WT mice (*P* < 0.05). Consistent with this enhanced inflammatory reaction, OVA-induced *Rheb1*-KO mice showed increased mucus production in their lungs (Fig. 3d), and the corresponding mucus production score of the *Rheb1*-KO mice was the highest (*P* < 0.05) (Fig. 3f). Immunohistochemical (IHC) staining of OVA-treated mice showed increased levels of α-SMA and Muc5ac compared with saline-treated WT mice, and the expression levels of α-SMA and Muc5ac were highest in the OVA-treated KO mice (Fig. 3g–j).

Based on all these data, we clarified that knockout of *Rheb1* in mouse myeloid cells would aggravate OVA-induced allergic asthma.

### *Rheb1* deletion induces Th2 cell response and inhibits Th1 cell response to OVA sensitization

**Figure 4:**
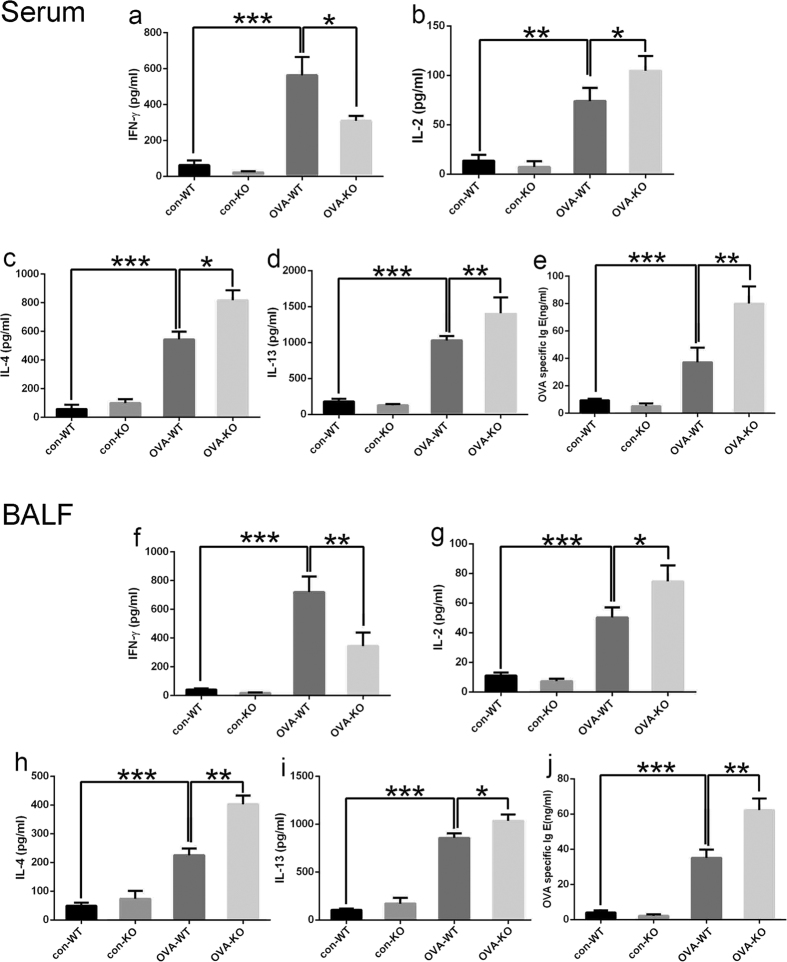
*Rheb1* deletion changed the cytokine levels and IgE level in serum and BALF. Cytokine levels in serum, expression of the Th1 cytokines IFN-γ (**a**), IL-2 (**b**) and the Th2 cytokines IL-4 (**c**), IL-13 (**d**). IgE levels in serum of each group (**e**). Cytokine levels in BALF, expression of the Th1 cytokines IFN- γ (**f**), IL-2 (**g**) and the Th2 cytokines IL-4 (**h**), IL-13 (**i**). IgE levels in BALF of each group (**j**). Gene expression data are shown as mean ± SD n ≥ 3; *P < 0.05; **P < 0.01; ***P < 0.001.

Th1 and Th2 cells secrete many cytokines that are involved in the pathophysiology of asthma. To determine the role of *Rheb1* deletion in secretion of these cytokines, we assessed the level of cytokines in serum and BALF of mice from both the control and asthma groups by ELISA. We found that some Th1 cytokines, IFN-γ and IL-2, and some Th2 cytokines, IL-4 and IL-13, were significantly elevated in serum and BALF from the OVA-challenged group compared with the control group. In addition, in the OVA-challenged group, levels of IFN-γ and IL-2 in KO mice were lower than those in WT mice (Fig. 4a,b,f,g), while levels of both IL-4 and IL-13 in serum and BALF of KO mice were higher than in WT mice (Fig. 4c,d,h,i). The spreading epidemic of allergies and asthma has heightened interest in IgE, the central player in the allergic response^25^. We also measured the level of IgE in serum and BALF. The level of IgE was increased in mice with OVA-induced allergic asthma, meanwhile, *Rheb1*-KO mice showed higher levels of IgE than all other groups (Fig. 4e,j).

Overall, our data show that OVA-challenged, *Rheb1*-KO mice displayed diminished production of Th1 cytokines, and enhanced production of Th2 cytokines and IgE levels compared with WT mice.

### *Rheb1* deletion in myeloid cells promotes M2 but inhibits M1 macrophage polarization

**Figure 5:**
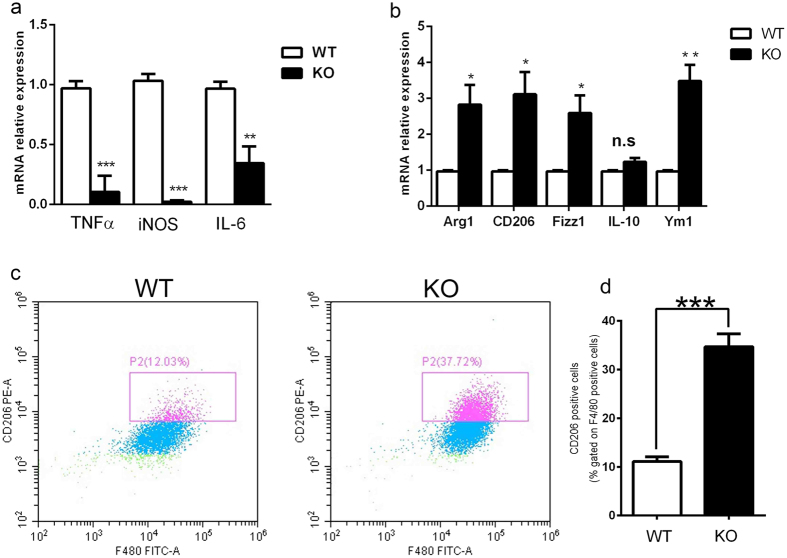
*Rheb1* deletion in myeloid cells promotes M2 but inhibits M1 macrophage polarization. (**a**) The mRNA expression levels of TNF-α, iNOS and IL-6 in WT and KO mice were determined by real-time PCR after LPS and IFN-γ stimulation for 6 h. (**b**) The mRNA expression levels of Arg1, CD206, Fizz1, IL-10 and Ym1 in WT and KO mouse bone marrow-derived macrophages (BMMs) were determined by real-time PCR after IL-4 stimulation for 48 h. (**c**) Representative results of flow cytometric analysis of IL-4-treated BMMs in WT and KO mice. The bar chart shows quantitative analysis of repeated results of flow cytometric analysis (**d**). Gene expression data are shown as mean ± SD n ≥ 3; *P < 0.05; **P < 0.01; ***P < 0.001; n.s. not significant.

To investigate whether *Rheb1* deletion in myeloid cells would affect macrophage polarization, we collected bone marrow cells from *Rheb1*-KO and WT mice at 4–6 weeks of age. Bone marrow derived macrophages (BMMs) were obtained from marrow as described in materials and methods. After LPS and IFN-γ stimulation, macrophages from *Rheb1*-KO mice showed obviously lower mRNA levels of TNF-α, iNOS and IL-6 in comparison with those of WT mice (Fig. 5a). Meanwhile after IL-4 stimulation, macrophages of *Rheb1*-KO mice displayed increased mRNA levels of Arg1, CD206, Fizz1, and Ym1, but no obvious changes in IL-10 (Fig. 5b). According to the results of flow cytometric analysis of IL-4-treated BMMs in WT and KO mice, the percentage of cells with double positive staining for F4/80 and CD206 among total BMMs was higher in *Rheb1-*KO mice than in WT mice (Fig. 5c,d). Consequently, these data indicated that *Rheb1-*KO in myeloid cells changed the polarization of macrophages, while macrophages from bone marrow of *Rheb1*-KO mice were hypersensitive to IL-4 stimulation and refractory to LPS and IFN-γ stimulation.

### *Rheb1*-KO mice displayed increased M2 polarization and decreased M1 polarization in BALF cells of mice with OVA-induced asthma

**Figure 6:**
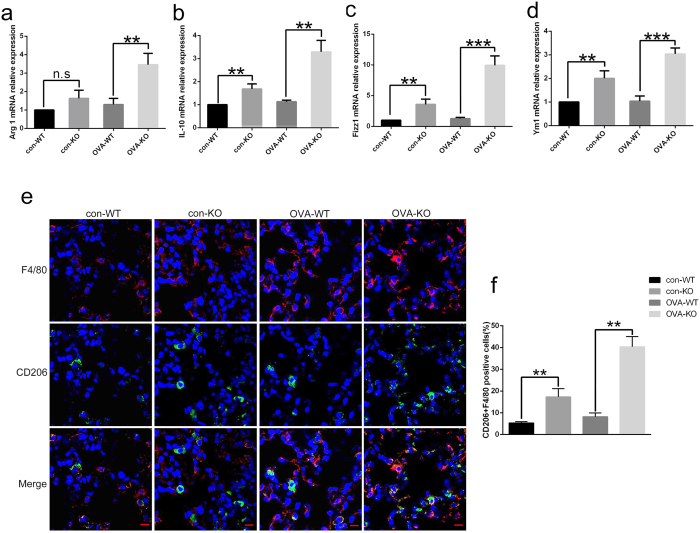
Analysis of alveolar macrophages’ M2 polarized state in WT and *Rheb1*-KO mice of each group. (**a–d**) The mRNA expression levels of the M2 genes Arg1, Fizz1, IL-10 and Ym1, from BALF cells of the control and asthma groups were determined by quantitative real-time PCR. (**e**) IF analysis of F4/80 (red) and CD206 (green) of lung cells from mice sensitized and challenged with saline or OVA. Images were captured with a confocal laser scanning microscope (Scale bar = 10 μm). (**f**) Quantitative analysis of F4/80 and CD206 positive cells by IF analysis. Gene expression data are shown as mean ± SD; n ≥ 3; **P < 0.01; ***P < 0.001.

**Figure 7:**
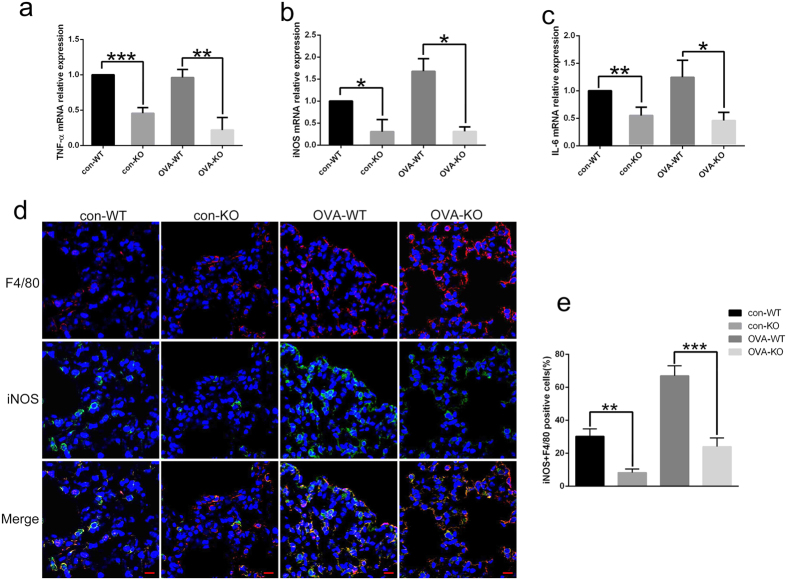
Analysis of alveolar macrophages’ M1polarized state in WT and *Rheb1*-KO mice of each group. (**a–c**) The mRNA expression levels of M1 genes TNF-α, iNOS and IL-6 in BALF cells of WT and KO mice, from control and asthma groups, was determined by real-time PCR. (**d**) IF analysis of F4/80 (red) and iNOS (green) of lung tissue of mice sensitized and challenged with saline or OVA (Scale bar = 10 μm). (**e**) Quantitative analysis of F4/80 and iNOS positive cells by IF analysis. Gene expression data are shown as mean ± SD; n ≥ 3; * means *P < 0.05; **P < 0.01; ***P < 0.001.

We also analyzed the polarization state of macrophages in BALF cells in WT and KO mice of the control and asthma groups. As shown in Fig. 6a–d, in the control group, the mRNA expressions of M2 markers such as Arg1, Fizz1, IL-10 and Ym1 in *Rheb1*-KO mice were greatly enhanced compared with those in WT mice, while in contrast, differences in mRNA expressions of M2 markers were more significant between *Rheb1*-KO mice and WT mice in the asthma group. IF staining of F4/80 and CD206 revealed that *Rheb1*-KO mice had higher expression of CD206 in F4/80 positive cells than WT mice, in both the control and asthma group. As expected, the difference was more marked in the asthma group than in the saline-treated group (Fig. 6e,f). Furthermore, the mRNA expression of M1 markers including IL-6, iNOS and TNF-α in *Rheb1*-KO mice was found to be reduced compared with that in WT mice (Fig. 7a–c). Moreover, IF staining showed that *Rheb1*-KO mice had lower expression of iNOS in F4/80 positive cells in comparison with WT mice, in both the asthma and control groups (Fig. 7d,e). We therefore concluded that *Rheb1* knockout in myeloid cells increases M2 polarization and decreases M1 polarization in macrophages, and these differences are increased in OVA-induced asthma.

## Discussion

In this study, to elucidate the function of *Rheb1* in allergic asthma and macrophage polarization, we first used myeloid-specific *Rheb1* deletion mice to create an allergic asthma model. We found that *Rheb1*-KO mice were more susceptible to OVA-sensitization and challenge than WT mice, and found increased activity of *Rheb1* and mTORC1 in myeloid cells of C57BL/6 mice with OVA-induced allergic inflammation. Moreover, M2 polarization was increased and M1 polarization decreased in alveolar macrophages of *Rheb1*-KO mice compared with WT mice. Based on these data, we inferred that *Rheb1* might participate in regulating macrophage polarization and mediating OVA-induced allergic asthma via an mTORC1-dependent signaling pathway.

Rapamycin, an mTOR inhibitor, is a clinically-approved drug used as an immunosuppressive agent that reduces organ transplant rejection^26^. Previous studies on allergic asthma induced by OVA in mice and rats suggested that rapamycin could attenuate inflammation, AHR, and mucous cell hyperplasia^27^. However, some other studies indicated that rapamycin has little effect on the inflammation and AHR of allergic airways^28^,^29^. Nevertheless, the results in our study are quite consistent compared with the uncertain effect of rapamycin on asthma in some previous studies, but are highly consistent with the effect of TSC1-KO macrophages in asthmatic mice. Considering this, we hypothesize that some conflicting effects of rapamycin on allergic asthma may be attributable to different cell types, different treatment approaches and drug doses, or the involvement of an mTORC1-independent pathway. In our study, as expected, *Rheb1* deletion in myeloid cells showed stable inhibition of mTORC1 and additionally contributed to M2 polarization of macrophages which caused serious inflammatory reactions in OVA-induced asthma.

Differing from the effects of mTORC1 induced by rapamycin in many other cell types, such as eosinophils, dendritic cells and T regulatory cells, the effect of reducing mTORC1 activity in macrophages is much more sustained, and in innate immunity macrophages are among the most abundant cells and one of the first to encounter allergens and other threats to homeostasis. Depending on the signals they receive, macrophages can undergo M1 or M2 polarization. Although the initial definition of these subtypes was largely on the basis of *in vitro* studies using bone marrow- or monocyte-derived macrophages^30^, there is now increasing evidence that these phenotypes also exist *in vivo*. M1 macrophages are induced by Th1 cytokines, particularly IFN-γ and LPS, and characteristically produce CXCL9 and CXCL10^31^,^32^. M1 macrophages typically participate in Th1 responses and modulate host defenses against intracellular pathogens, tumor cells, and tissue debris^33^. By contrast, M2 macrophages are induced by IL-4 and IL-13 and other Th2 cytokines, and they typically produce chemokines such as CCL17, CCL22, and CCL24^31^,^34^,^35^. Recent studies have identified roles for M2 macrophages in models of allergic inflammation of the airways^36^,^37^. Despite the evidence for different macrophage phenotypes, emerging studies have demonstrated the phenotypic plasticity of macrophages and the functional overlap between subtypes of these cells^30^,^38^. For example, in an infectious model of *Listeria*, circulating monocytes can first have an M1 phenotype but subsequently develop an M2 phenotype. Moreover, recent human studies suggest that M1 pulmonary macrophages play a key role in the development of severe asthma. Goleva *et al*.^39^ showed that steroid-resistant asthmatic patients have increased expression of M1 and decreased expression of M2 markers on macrophages in BALF. These observations highlight the complex roles of pulmonary macrophages in the regulation of the pathogenesis of severe asthma. Therefore, we were interested in the roles of these macrophage subtypes in OVA-induced asthma. M2 polarization is mainly mediated by inhibition of the mTORC1 pathway, so *Rheb1* may regulate a key checkpoint of the mTORC1 pathway in macrophage polarization^16^. In this study, we concluded that *Rheb1* deletion in myeloid cells reorganizes the expression of Th1 and Th2 cytokines. We also found that in *Rheb1*-KO mice, levels of TNF-α, iNOS and IL-6, markers of M1-polarized macrophages, were significantly decreased, but Arg1, CD206, Fizz1, and Ym1, markers of M2-polarized macrophages, were increased, as detected by quantitative PCR. In addition, flow cytometry analysis revealed that, in comparison to WT mice, *Rheb1*-KO mice were more susceptible to IL-4 stimulation. Meanwhile, immunofluorescence analysis showed that when *Rheb1*-KO mice were challenged by OVA, they expressed more CD206 in F4/80 positive cells, but displayed lower levels of iNOS expression in F4/80-positive macrophages compared with those from OVA-challenged WT mice. It is known that *Rheb1* is a direct downstream target of the *TSC1/2* complex and also an upstream regulator of mTORC1, acting to regulate translation and cell growth^40^. A central role of mTOR in integrating cytokine signaling and regulating T effector lineage commitment and immune responses has been emphasized^41^. In view of these findings, we inferred that *Rheb1* as a downstream effector of the *TSC1/2* complex can directly participate in regulating macrophage polarization and influencing OVA-induced allergic asthma by reorganizing Th1/Th2 cytokine expression, which may be dependent on the TSC–mTORC1 signaling pathway. All in all, deletion of *Rheb1* in myeloid cells, by directly inhibiting *Rheb1* GTPase and mTORC1 activity, blocks M1 polarization and promotes M2 polarization, thus *Rheb1* in macrophages may be one of the key control points of asthma.

In summary, we demonstrated that *Rheb1*-KO directly suppresses M1 and promotes M2 polarization of macrophages in an mTOR-dependent manner, and showed that its essential role in M2 activation, as well as in regulating allergic asthma, is mainly mediated by inhibiting the mTOR pathway. In addition, M2 polarization of macrophages contributes to aggravating the inflammatory reaction of OVA-induced allergic asthma. Thus, *Rheb1* regulates a key checkpoint in macrophage polarization and the inflammatory reaction of allergic asthma, and *Rheb1*-KO in macrophages results in increased inflammatory disorders. This study highlights potential and critical targets for macrophage-directed therapeutic strategies for controlling allergic inflammatory disease.

## Methods

### Animals and care

C57BL/6 mice were used in this study. Lys-MCre mice were purchased from The Jackson Laboratories (Bar Harbor, ME, USA, stock NO. 004781). *Rheb1*flox/flox mice were kindly gifted by Professor Bo Xiao, Sichuan University. The mice were housed individually under standard conditions of temperature and humidity on a 12-h light/dark cycle. Lys-MCre mice were first mated with *Rheb1*flox/flox mice to generate Lys-MCre-*Rheb1*flox/+mice. Their offspring were then backcrossed to homozygote floxed mice (Lys-MCre-*Rheb1*flox/+X *Rheb1*flox/flox) to generate Lys-MCre-*Rheb1*flox/flox mice, hereafter, Lys-MCre-*Rheb1*flox/flox mice are referred to as homozygote KO mice and *Rheb1*flox/flox as wild-type (WT) mice. All animals were cared for according to the guidelines of the Southern Medical University Animal Care and Use Committee. All experimental protocols were approved by the Southern Medical University Animal Care and Use Committee.

We performed genotyping using genomic DNA isolated from mouse tail biopsies, and the primers are listed below:

Lys-MCre common: 5′-CTT GGG CTG CCA GAA TTT CTC3′; mutant: 5′-CCC AGA AAT GCC AGA TTA CG-3′; WT: 5′-TTA CAG TCG GCC AGG CTG AC-3′.

*Rheb1*-Forward: 5′-GCC CAG AAC ATC TGT TCC AT-3′; *Rheb1*-Reverse: 5′-GGT ACC CAC AAC CTG ACA CC-3′.

### OVA-induced allergic inflammation

Mice aged 6 to 8 weeks were sensitized and challenged with OVA to induce allergic inflammation. In brief, all experimental mice were injected intraperitoneally (i.p.) with 20 μg OVA (Grade V, Sigma-Aldrich, St. Louis, MO, USA) mixed with 2 mg aluminum hydroxide (Aladdin Biotech, Xi’an, China) in 0.2 mL of saline on day 0 and day 7. The mice in the control group received 0.2 mL of saline. From day 24 to day 30, the OVA-sensitized mice were challenged with 2% aerosolized OVA, and the control group mice were challenged with saline for 30 min per day^42^,^43^.

### Assessment of airway hyper-responsiveness

Methacholine-induced airway reactivity was assessed 24 h after the final aerosol challenge by direct plethysmography (Buxco Electronics, Wilmington, NC, USA). Mice in each group were exposed to a series of incremental doses of aerosolized methacholine (3.625, 6.25, 12.5, 25, 50, 100 mg/mL) for 2 minutes at each concentration, and airway hyper-responsiveness was recorded as enhanced pause (Penh), which was calculated using the program provided by Buxco Electronics. Results are expressed as the maximal resistance after each dose of methacholine minus baseline (PBS alone) resistance.

### Collection of bronchoalveolar lavage fluid (BALF)

One day after the last challenge, mice were injected intraperitoneally (i.p.) with 8 μL/g of 1% pentobarbital. The lung was then lavaged twice with 0.5 mL of cold PBS. The collected BALF was centrifuged at 1000 × *g* at 4 °C for 5 minutes, the supernatants were stored at −80 °C for enzyme-linked immunosorbent assay (ELISA) and the pellet was used for differential cell counting, quantitative RT-PCR and western blot analysis.

### Differential cell counting

The cell pellets harvested from BALF were resuspended in 100 μL PBS and centrifuged onto slides, then stained with Wright–Giemsa stain for 6 min. Two independent, blinded investigators counted the cells using a microscope. Approximately 200 cells were counted in each of four different random locations.

### Quantitative RT-PCR

Total RNA was isolated with Trizol (Life Technologies, Carlsbad, CA, USA) and reverse transcription was performed with reverse transcriptase according to the manufacturer’s instructions. RT-PCR was performed using multiple kits (SYBR Premix Ex TAQ, Drr041a, Takara Bio, Shiga, Japan) according to the manufacturer’s instructions. To determine the relative expression level of mRNA, each gene was normalized to the expression level of the housekeeping gene *GAPDH*. The primers used in the present study are listed below: Arg-1, forward, 5′-ACCTGGCCTTTGTT GATGTCCCTA-3′, reverse, 5′-AGAGATGCTTCCAACTGCCAGACT-3′; CD206, forward, 5′-TTGGACGGATAGATGGAGGG-3′, reverse, 5′-CCAGGCAGTTGAG GAGGTTC-3′; Fizz-1, forward, 5′TCCAGCTGATGGTCCCAGTGAATA-3′, reverse, 5′-ACAAGCACACCCAGTAGC AGTCAT-3′; Ym1, forward, 5′-AGAAGGGA GTTTCAAACCT-3′, reverse, 5′-GTCTTGCTCATG TGTGTAAGTGA-3′; IL-10, forward, 5′-GCTCTTACTGACTGGCATGAG-3′, reverse, 5′-CGCAGCT CTAGGAGCATGTG-3′; TNF-α, forward, 5′-GAGTGACAAGCCTGTAGCC-3′, reverse, 5′-CTCCTGGTATGAGATAGCAAA-3′; iNOS, forward, 5′-CACCAAG CTGAACTTGAGCG-3′, reverse, 5′-CGTGGCTTTGGGCTCCTC-3′; IL-6, forward, 5′-ACAAAGCCAGAGTCCTTCAGAGAG-3′, reverse, 5′-TTGGATGGTC TTGGTCCTTAGCCA-3′; GAPDH, forward, 5′-TCAACGACCCCTTCATTGAC-3′, reverse, 5′-ATGCAGGGATGATGTTCTGG-3′.

### Enzyme-linked immunosorbent assay (ELISA)

Cytokine levels (IL-2, IL-4, IFN-γ, IL-13, IgE) in BALF and serum were measured using specific kits (Elabscience, Wuhan, China) according to the protocols provided by the manufacturer. All measurements were performed in duplicate and all sample measurements were performed without any additional modification. The results were calculated using Excel 2010 (Microsoft Office, Microsoft, Redmond, WA, USA).

### Histological assessment

The lung tissues of each mouse were fixed in 4% paraformaldehyde at 4 °C for 24 hours, then the fixed tissues were embedded in paraffin and cut into 3-μm sections with a microtome (Leica, Nussloch, Germany). The areas of inflammation and mucus production were analyzed by hematoxylin/eosin (H&E) and periodic acid-Schiff (PAS) staining, respectively. The stained slides were observed under a light microscope. Quantitative analyses of inflammatory cells and goblet cells in lung tissues were performed in a blinded fashion as previously described^26^,^44^. Briefly, an inflammation score was used to assess the severity of infiltration, based on a five-point scoring system: 0, normal; 1, a few cells; 2, a ring of cells 1 cell deep; 3, a ring of cells 2 to 4 cells deep; and 4, a ring of cells >4 cells deep. A mucus score was used to evaluate the extent of mucus secretion using a five-point grading system: 0, no goblet cells; 1, <25% goblet cells; 2, 25–50% goblet cells; 3, 50–75% goblet cells; and 4, >75% goblet cells. The scores were calculated from at least three different fields for each lung section, and a mean score was obtained from three animals.

### Immunohistochemistry (IHC)/immunofluorescence (IF) staining

For IHC, slides were incubated with the primary antibodies a-SMA (Cell Signaling Technology, Danvers, MA, USA; 1:100) or anti-Muc5ac (Abcam, Cambridge, MA, USA; 1:100) overnight at 4 °C, then the slides were washed with PBS and incubated with the rabbit anti-mouse HRP secondary antibody (Cell Signaling Technology, Inc., Danvers, MA, USA; 1:2000) for 1 h at room temperature. The sections were then washed in PBS, and visualization was performed with the chromogen diaminobenzidine (Zsbio, Beijing, China) in solution followed by counterstaining with hematoxylin solution. All sections were observed and photographed under an Olympus microscope. For IF, we incubated slides with the primary antibodies F4/80 (Santa Cruz Biotechnology, Santa Cruz, CA, USA; 1:100), phospho-S6 ribosomal protein (Ser235/236) (Cell Signaling Technology, Inc.; 1:200), CD206 (Biolegend, San Diego, CA, USA; 1:100) and iNOS (Abcam, Cambridge, UK; 1:100) overnight at 4 °C, then the slides were washed with PBS and incubated with the following secondary antibodies for 1 hour at room temperature: Alexa Fluor 594 donkey anti-mouse IgG1 (Life Technologies, Carlsbad, CA, USA), Alexa Fluor 488 goat anti-mouse IgG2b (Life Technologies). The slides were then washed with PBS three times, and nuclei were counterstained with DAPI. Images were obtained using a confocal laser scanning microscope (Olympus, Tokyo, Japan). F4/80 was labelled in red and the rest were labelled in green; nuclei were stained blue.

### Cell preparation

To obtain bone marrow (BM)-derived macrophages, BM was isolated from 4–6 week-old mice; after erythrocyte lysis, mononuclear cells were incubated with M-CSF (10 ng/mL) in RPMI1640 (Gibco, Germany) supplemented with 10% FBS (Gibco, Germany) for 5 days as described^45^. The medium was changed every 2 days. For M1 or M2 polarization, cultured BM-derived macrophages (BMMs) were incubated with IFN-γ (20 ng/mL) and LPS (100 ng/mL) for 6 hours or with IL-4 (20 ng/mL) for 48 hours.

### Macrophage function assay

Peritoneal macrophages were collected from intraperitoneal injection of Thioglycollate Broth (Sigma #70157) into mice. The cells were suspended in 1640 medium and cultured at 10^4^ cells per well in 96-well plates. For India ink uptake assay, after cells adhere to the plates, India ink (10 ul/mL) was added to the culture plates, which was then incubated for 4 h. The cells were observed with microscope and counted for analysis. Macrophages phagocytosis also evaluated by neutral red resorption. 200 μl neutral red solutions were added and cells were incubated for 1 hr. Supernatant was discarded and cells were washed twice in PBS to remove the neutral red that was not phagocytized by macrophages. Then, cell lysate solution (ethanol and 0.01% acetic acid at the ratio of 1:1) was added to lysed cells. Next, cells were incubated at 4 °C, overnight. The optical density was measured at 490 nm by a microplate reader.

### Flow cytometry

Different cells types in blood, bone marrow and BALF from KO and WT mice were analyzed by flow cytometry. Cells were incubated with FITC-CD11b (eBioscience, San Diego, CA, USA), PC5.5-B220 (BD PharMingen, San Diego, CA, USA) and 780-CD3 (eBioscience) to detect monocytes; with FITC-CD11b and APC-F4/80 (eBioscience) to detect macrophages; with FITC-CD11b and PE-ly6G (eBioscience) to detect neutrophils; with FITC-CD11b, APC-CD11c (eBioscience) and PC5.5-B220 to detect dendritic cells and with FITC-CD11b and APC-Gr-1 (eBioscience) to detect MDSCs. BMMs treated with IL-4 (20 ng/mL) for 48 h were collected, and then incubated with APC-F4/80 (eBioscience) and FITC-CD206 (BioLegend) antibodies at 4 °C for 30 minutes. Cells were analyzed on a flow cytometer (Beckman Coulter, Brea, CA, USA) with Cytexpert software. The experiment was repeated three times.

### Western blotting analysis

Protein samples were extracted from bone marrow-derived macrophages and the alveolar macrophages sorted by flow cytometry (CD11B^+^ F4/80^+^) obtained from BALF. Cells were lysed in lysis buffer containing 2% sodium dodecyl sulfate with 2 M urea, 10% glycerol, 10 mM Tris–HCl (pH 6.8), 10 mM dithiothreitol and 1 mM phenylmethylsulfonyl fluoride. Proteins were separated by 10% sodium dodecyl sulfate-polyacrylamide gel electrophoresis. After electrophoresis, the proteins were transferred to membranes (Bio-Rad Laboratories, Berkeley, USA) by the wet transfer method. Each membrane was blocked with TBST (100 mM Tris–HCl pH 7.5, 150 mM NaCl, 0.05% Tween20) with 5% non-fat dried milk for 1 h at room temperature, and then incubated with primary antibodies overnight on a shaker at 4 °C. The appropriate HRP-coupled secondary antibody was then added, and incubated for 1 h at room temperature. After the membranes were treated with enhanced chemiluminescence western blot detection reagents (ECL Kit, Amersham Biosciences, Piscataway, NJ, USA), then the binding of specific antibodies was detected by chemiluminescence. β-actin was used as a protein loading control.

### Statistical analysis

All statistical analyses and graphing were carried out using GraphPad Prism software (GraphPad Software). Comparisons between two groups were performed with Student’s t-test for unpaired variables. Data are reported as means ± SD unless otherwise specified. *P*-values < 0.05 were considered statistically significant.

## Additional Information

**How to cite this article**: Li, K. *et al*. *Rheb1* deletion in myeloid cells aggravates OVA-induced allergic inflammation in mice. *Sci. Rep.* **7**, 42655; doi: 10.1038/srep42655 (2017).

**Publisher's note:** Springer Nature remains neutral with regard to jurisdictional claims in published maps and institutional affiliations.
